# XUV fluorescence as a probe of interatomic coulombic decay of resonantly excited He nanodroplets

**DOI:** 10.1038/s41598-025-34677-x

**Published:** 2026-01-09

**Authors:** Keshav Sishodia, Ltaief Ben Ltaief, Niklas Scheel, István B. Földes, Andreas Hult Roos, Martin Albrecht, Matyáš Staněk, Lucie Jurkovičová, Ondrej Hort, Jaroslav Nejdl, Ernesto García-Alfonso, Nadine Halberstadt, Jakob Andreasson, Eva Klimešová, Maria Krikunova, Sivarama Krishnan, Andreas Heidenreich, Marcel Mudrich

**Affiliations:** 1 Quantum Center of Excellence for Diamond and Emergent Materials, School of Interdisciplinary Sciences and Department of Physics, Indian Institute of Technology Madras, Chennai, 600036 India; 2 ELI Beamlines facility, The Extreme Light Infrastructure ERIC, Za Radnicí 835, Dolní Břežany, 253 41 Czech Republic; 3 Department of Physics and Astronomy, Aarhus University, Ny Munkegade 120, Aarhus C, 8000 Denmark; 4 HUN-REN Wigner Research Centre for Physics, Budapest, H-1121, Hungary; 5 Czech Technical University in Prague, FNSPE, Prague, 115 19 Czech Republic; 6 Laboratoire Collisions, Agrégats, Réactivité (LCAR), Université de Toulouse, CNRS, Toulouse, 31062 France; 7 Wildau Technical University of Applied Sciences, Hochschulring 1, Wildau, 15745 Germany; 8 Kimika Fakultatea, Euskal Herriko Unibertsitatea (UPV/EHU) and Donostia International Physics Center (DIPC), P. K. 1072, Donostia, 20080 Spain; 9 IKERBASQUE, Basque Foundation for Science, Bilbao, 48011 Spain; 10 Institute of Physics, University of Kassel, Kassel, 34132 Germany

**Keywords:** Materials science, Nanoscience and technology, Physics

## Abstract

Superfluid He nanodroplets resonantly excited by extreme ultraviolet (XUV) pulses can relax via interatomic coulombic decay (ICD), generally considered an ultrafast process. Here, we introduce a novel approach to probe the dynamics of ICD in He nanodroplets over timescales ranging from femtoseconds to nanoseconds. Our method relies on detecting XUV fluorescence emitted from the nanodroplets as they are driven into a nanoplasma by subsequent intense infrared pulses. Nanoplasma ignition is facilitated by tunnel ionization of XUV-excited He$^*$ atoms attached to the droplets; it thus serves as a sensitive probe of their relaxation dynamics. The observed nanosecond-scale decay is attributed to ICD between pairs of He$^*$ atoms undergoing roaming motion on the droplet surface, as supported by quantum-mechanical and classical model calculations.

## Introduction

In weakly bound condensed-phase systems such as Van der Waals complexes, liquids, and soft matter, local excitations or inner-shell ionizations can relax by non-local, intermolecular mechanisms. A particularly important instance is interatomic or intermolecular coulombic decay (ICD), in which energy deposited in one atom or molecule is transferred to a neighboring site, leading to its ionization^1^. ICD was first predicted to occur in inner-valence ionized molecular clusters by Cederbaum et al.^2^. Following pioneering experimental studies mainly on small rare-gas atomic clusters and molecular complexes^3–5^, it was recognized that ICD is generally relevant to a wide range of weakly bound systems irradiated by energetic photons or charged particles, including liquids^6,7^, solids^8^, quantum dots^9^, and biological matter^1,10,11^. Although ICD is a non-local process, it can be highly efficient mainly owing to the ultrafast timescales – typically femtoseconds to picoseconds – on which it proceeds. Here, we investigate ICD in superfluid He nanodroplets initiated by their resonant excitation using short extreme ultraviolet (XUV) pulses. He nanodroplets have turned out to be particularly well-suited model systems for ICD studies due to their exceptional properties – extremely weak binding of the constituent He atoms resulting in a homogeneous superfluid density distribution, and a simple electronic structure of He resulting in highly resolved electron spectra^12^. The main result of the present combined experimental and theoretical study is that ICD in He nanodroplets proceeds both on ultrafast ($<1$ ps) and on much longer timescales ($>1$ ns). The latter is attributed to a roaming motion of localized He excitations on the surface of the superfluid He nanodroplets prior to ICD.

Resonant excitation of superfluid He nanodroplets initiates an intricate coupled electronic and nuclear relaxation dynamics^13–15^. After localization of the excitation on a metastable He$^*$ atom, within $\lesssim 1$ ps a void cavity (‘bubble’) with radius $R=0.68$ nm forms around the He$^*$ atom^14,16–18^. Due to the repulsive interaction between the He$^*$ and the surrounding He atoms, the localized He$^*$ subsequently emerges to the droplet surface from which it is either ejected into the vacuum or remains loosely bound and roams about the droplet surface. The situation changes when multiple He$^*$ are excited in one He nanodroplet at high photon flux or in large droplets. At high density of He$^*$ excitations as reached using intense free-electron laser pulses, nearest-neighbor pairs of He$^*$ are present in the droplets with short interatomic separations $\lesssim 1~$nm^19^. These pairs can decay by ICD according to the reaction

1 $$\begin{aligned} \textrm{He}^* + \textrm{He}^* \rightarrow \textrm{He} + \textrm{He}^+ + e_\textrm{ICD}, \end{aligned}$$

as predicted by Kuleff et al. for multiply excited clusters^20^. Under these conditions, ICD is an ultrafast process that occurs within $\lesssim$ 1 ps as it is driven by the He fluid dynamics of the two bubbles forming around the He$^*$ and merging into one^19^.

ICD processes of the type given in Eq. (1) have also been observed in a different scenario. When a He nanodroplet or a heavier rare-gas cluster is massively ionized by an intense infrared pulse^21,22^ or x-ray pulse^23,24^, excited states of the constituent atoms and ions are populated by electron-ion recombination in the expansion phase of the cluster. These ICD-related processes manifest as characteristic features in electron spectra that are superimposed on a broad distribution of nanoplasma electrons^21–23^. In a time-resolved experiment investigating the ultrafast evolution of a xenon nanoplasma induced by an intense X-ray pulse, the fast decay ($\sim 250$ fs) of the excited-state population was ascribed to ICD^24^.

In the case of low photon fluence and large He droplets as in the present experiment, the He$^*$ relax to the 1s2s $^1$S long-lived state and emerge to the droplet surface prior to ICD^13–15^. This is evidenced by sharp lines in the electron spectra and fully isotropic electron angular distributions measured using similarly low-intensity synchrotron radiation^25,26^. Thus, this type of ICD of He$^*$ occurring at the droplet surface is expected to be a slow process that proceeds on a timescale $\gg 1$ ps after resonant excitation of the He droplet. In the present work, we track this ICD process (Eq. 1) in time under conditions of resonant excitation of large He droplets by low-fluence extreme ultraviolet (XUV) pulses.

To monitor the decay dynamics of the excited He$^*$ atoms attached to the droplets, we introduce a novel probing scheme: an intense near-infrared (NIR) laser pulse, delayed with respect to the XUV excitation, terminates the evolution of the He$^*$ excited in the He nanodroplets by inducing strong-field ionization and thus transforming the droplets into a nanoplasma. The resulting fluorescence emission from excited atomic and ionic states is then detected by an XUV spectrometer. These states are populated by electron-ion recombination in the expanding nanoplasma. NIR-induced strong-field ionization of large molecules and clusters is highly sensitive to the presence of free or quasi-free electrons that act as seeds to initiate the ionization avalanche. Therefore, the fluorescence emission yield is indicative of the population of He$^*$ attached to the He nanodroplets; He$^*$ are easily tunnel ionized by the intense NIR laser pulse due to their much lower ionization energy ($E_i^\mathrm {He^*}=4$ eV) as compared to ground-state He atoms ($E_i^\textrm{He}=24.6$ eV). In addition to XUV fluorescence measurements, we have also detected photoelectron spectra from He nanodroplets under the same XUV irradiation conditions to evidence the presence of He$^*$ in the excited nanodroplets and their decay by ICD.

Recently, Sumfleth et al. have presented fluorescence emission spectra from laser-induced He nanoplasmas, but no time-resolved measurements were reported^27^. In those experiments, mainly a series of emission lines from excited states of the He$^+$ ion were observed. Our experiment combines that method with XUV pump and NIR probe time-resolved probing of the He nanodroplet dynamics. This approach resembles an experiment carried out by Medina et al. with a similar setup; there, the pump-probe dynamics of photoionized He nanodroplets was studied by monitoring the rate of NIR-induced avalanche ionization^28^. In that experiment, a long-lasting persistent enhancement of the avalanche ionization probability was found; in contrast, here we observe a drop of the nanoplasma fluorescence signal on a time scale of about 2-9 ns, with a systematic dependency on the He nanodroplet size. These findings are rationalized by quantum mechanical simulations of the structure of He$^*$ binding to the He droplet surface, classical simulations of the motion of He$^*$ atoms at the droplet surface, and classical model calculations of the nanoplasma ignition process. The observed drop of nanoplasma fluorescence is attributed to the decay of pairs of He$^*$ atoms by ICD, mediated by a slow roaming motion of the He$^*$ about the He droplet surface as a consequence of the low temperature and the superfluid nature of He nanodroplets.

## Experimental setup

**Fig. 1:**
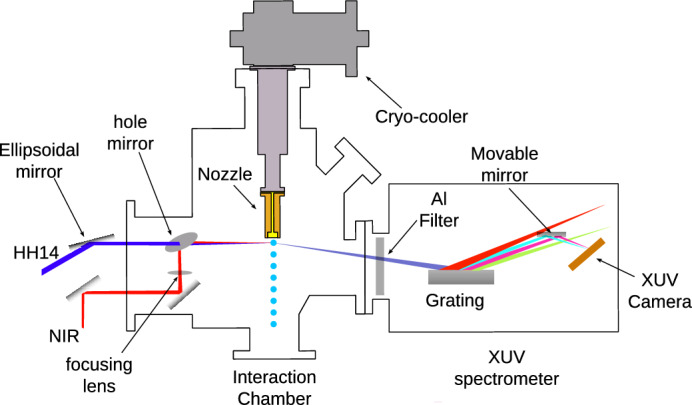
Schematic representation of the experimental setup. The He droplet source is mounted directly into the interaction chamber to take advantage of a high density of He droplets. The NIR and XUV beams are focused and superimposed onto the droplet beam in the interaction region just 1 mm downstream of droplet source. The XUV spectrometer is mounted next to the interaction region in the direction of propagation of the XUV beam.

We performed the experiment at the MAC end-station of the high harmonic generation (HHG) Beamline at the Extreme Light Infrastructure (ELI) Beamlines located in Dolní Břežany, Czech Republic^29^. Figure 1 shows a schematic representation of the experimental setup. A beam of He nanodroplets was generated by continuous supersonic expansion of He gas at a stagnation pressure of 50 bar out of a cryogenic nozzle with a diameter of 5 $\mu$m cooled to a temperature ranging between 11 K and 14.5 K. This corresponds to an average size of He droplets between $\left\langle N \right\rangle = 1\times 10^{4}$ and $5\times 10^{5}$ He atoms per droplet ($R=5$-17 nm, respectively). The mean droplet sizes are determined based on the nozzle temperature by comparison with previous measurements^30^. The XUV and NIR pulses were focused in the center of the interaction region at a small angle with respect to each other.

The synchronized XUV and NIR pulses were generated by a commercial femtosecond laser system operating at a repetition rate of 1 kHz. It provides NIR pulses with energies up to 12 mJ and a pulse duration of $\le 35$ fs centered at a wavelength of 800 nm. The laser beam was split into an intensity ratio of 8:2, where 80 % of the beam was guided to the HHG Beamline that generates XUV pulses^31^ and 20% of the beam was guided toward the MAC endstation.

In the HHG Beamline, the second harmonic of the NIR beam was generated by passing the beam through a beta-barium borate (BBO) crystal. The resulting UV beam was focused near the orifice of a pulsed valve from which Kr gas was expanded into the vacuum at a repetition rate of 500 Hz. The pressure of the Kr gas and the focusing conditions of the second harmonic beam were optimized to generate the highest intensity of the 14$^{\text {th}}$ harmonic (HH14) of the NIR at a photon energy of 21.8 eV^32^. This single harmonic HH14 was selected by a grating monochromator^31^. In the interaction region in MAC chamber, the XUV photon fluence $\Phi$ was measured using a calibrated XUV-sensitive photodiode yielding $\approx 5\times 10^6$ photons/pulse. The XUV pulse had a duration of $\approx 50$ fs, an energy bandwidth (FWHM) of 76 meV and was focused to a spot size (FWHM) of $64\times 47~\mu$m$^{2}$ using an ellipsoidal mirror, yielding a peak intensity of $1.5\times 10^7$ Wcm$^{-2}$.

The weaker NIR beam (20 %) passed through a delay line with a mechanical translation stage that can move up to 1 m with a minimum step size of 20 nm, allowing us to control the delay between the XUV and the NIR pulses between -0.6 ns and 6 ns. Due to dispersion in the optical elements, air path of the NIR beam, and partial compression by the chirped mirrors, the NIR pulse duration was about 150 fs in the interaction region. The pulse energy of the NIR pulse was attenuated to 180-400 $\mu$J. The NIR pulse was focused to a spot size of $18\times 43~\mu$m$^{2}$ using a focusing lens with a focal length of 150 mm, yielding a peak intensity of $10^{14}$-$10^{15}$ Wcm$^{-2}$. A mirror with a central bore hole allowed us to recombine the two beams in a nearly collinear geometry in the interaction region at a distance of about 1 mm from the orifice of the cryogenic nozzle that generated the He nanodroplets. The positions of the two beam foci were determined by imaging them on a YAG:Ce fluorescence screen that was inserted into the focal plane after retracting the cryogenic nozzle from the center of the interaction chamber.

An XUV spectrometer was mounted in the direction of propagation of the XUV beam behind the interaction region to detect the fluorescence signal excited by the XUV and NIR beams in He droplets. The XUV spectrometer consisted of a concave grating with 1200 grooves/mm that dispersed the fluorescence light and focused individual spectral lines. A selected spectral region was imaged on an XUV camera by a movable mirror. The XUV spectrometer provides a resolution of 0.05 nm^33^.

To support our investigation using fluorescence spectroscopy, we also measured photoelectron spectra from He droplets under the same conditions in another experimental run. In that experiment, a magnetic bottle electron spectrometer (MBES) was mounted at the MAC endstation^34,35^ and the droplet beam was skimmed twice to produce a low-density stream of droplets for photoelectron spectroscopy. The MBES contains a 2 m long flight tube and provides a high electron collection efficiency and an energy resolution of $\Delta E/E \approx 5\%$^36^. In our experiments, the energy resolution was limited by the bandwidth of the XUV pulse.

## Results and discussion

### Nanoplasma XUV fluorescence spectra and photoelectron spectra

**Fig. 2:**
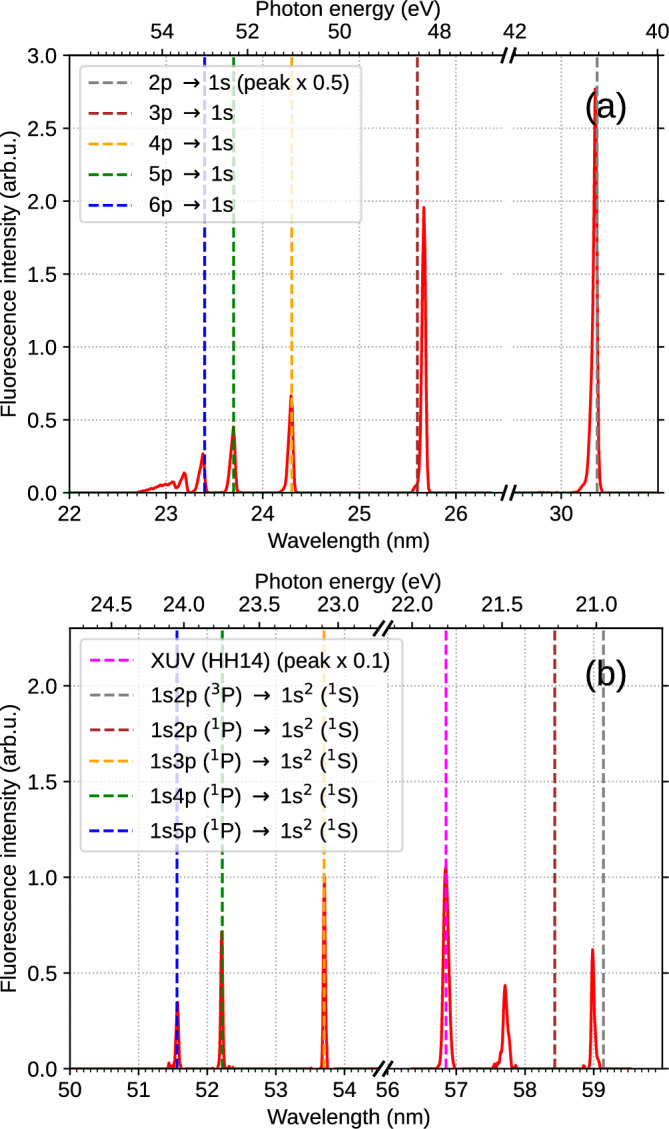
Fluorescence spectra emitted from helium nanoplasma containing $3\times 10^{4}$ atoms. Droplets were excited by the XUV pulse and ignited to the nanoplasma state through avalanche ionization by the temporally delayed NIR pulse with a pulse energy of 230 $\mu$J. (a) Spectral lines attributed to excited He ions, He$^{+*}$. (b) Spectral lines attributed to excited neutral He atoms, He$^{*}$. The dashed vertical lines show the positions of atomic emission lines from the literature^37^. The dashed magenta line shows the spectrum of the 14$^{\text {th}}$ harmonic generated in the HHG beamline.

Typical spectra of the fluorescence emitted by He droplets after ignition of a nanoplasma by the NIR pulse are shown in Fig. 2. The strongest fluorescence is observed in the form of emission lines of the excited He ion, He$^{+*}$, see Fig. 2a. These lines are identified as the transitions 2p$\rightarrow$1s at 30.37 nm, 3p$\rightarrow$1s at 25.6 nm, 4p$\rightarrow$1s at 24.3 nm, 5p$\rightarrow$1s at 23.7 nm and 6p$\rightarrow$1s at 23.4 nm^38^. This observation matches the one by Sumfleth et. al.^27^ who reported fluorescence spectra in the spectral regions corresponding to the 6p$\rightarrow$1s transition and from higher excited states up to the ionization threshold of He$^{+}$ ions for large He droplets containing $1.2\times 10^6$ atoms. Highly excited He$^{+*}$ cations are populated by recombination of electrons with He$^{2+}$ dications, which subsequently decay by fluorescence. Additionally, recombination of electrons with He$^+$ monocations in the nanoplasma leads to the formation of excited neutral He$^{*}$ atoms. The fluorescence emitted by He$^{*}$, shown in Fig. 2b, results from atomic transitions 1s3p $\rightarrow$ 1s$^{2}$ at 53.7 nm, 1s4p $\rightarrow$ 1s$^{2}$ at 52.2 nm and 1s5p $\rightarrow$1s$^{2}$ at 51.5 nm^38^. The total fluorescence yield from He$^{+*}$’s was about six times higher than that from He$^{*}$’s, indicating that a large fraction of He atoms in the nanoplasma are transiently doubly ionized (‘inner ionization’)^39^. Subsequent recombination into He$^{+*}$ monocationic states is highly efficient, whereas recombination into neutral He$^{*}$ states remains infrequent, in line with previous observations of the emission of high yields of electrons and He$^+$ ions from the nanoplasma^40,41^. The fluorescence spectrum of He$^{*}$’s also contains the spectral line of the XUV pump pulse at 56.8 nm.

**Fig. 3:**
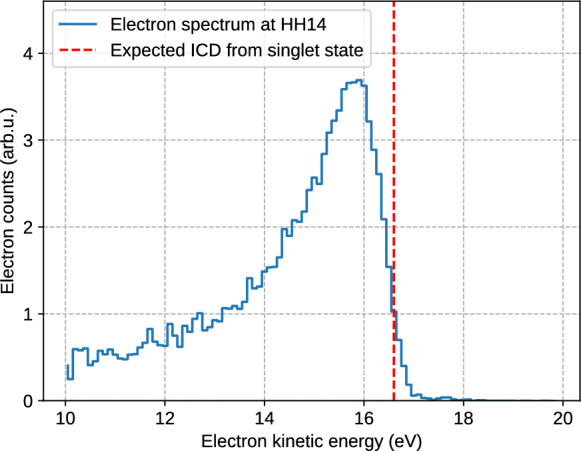
Electron spectrum recorded for He nanodroplets containing $5\times 10^5$ He atoms (mean radius $R\approx 17$ nm) irradiated by an XUV pulse with a photon energy of 21.8 eV. The peak around 15.55 eV manifests the emission of electrons by ICD of multiple He excitations per He droplet. The dashed line indicates the expected electron energy for ICD of pairs of unperturbed He$^*$ atoms in the 1s2s $^1$S state.

The corresponding photon energy of the XUV pump pulse, 21.8 eV, nearly matches the maximum of the main absorption band of He droplets, correlated to the 1s2p $^1$P state of He. At this photon energy, the absorption cross section $\sigma$ is estimated to 15 Mb per He atom^42,43^. For a droplet with a mean radius $\langle R\rangle =9$ nm containing $\langle N\rangle = 7\times 10^4$ He atoms, we estimate a mean number of excitations per droplet of $\Phi \sigma \langle N\rangle \approx 0.17$ He$^*$ and the probability of double excitations is $\sim 1.5$ % according to Poissonian statistics. Figure S1 shows the excitation probability as a function of mean droplet size, see the Supplementary Material for details. Therefore, any dynamics due to interacting He$^*$ in one droplet can be safely ascribed to pairs of He$^*$, while the contribution of three or more He$^*$ in one droplet is negligible. With the XUV beam intersecting the He droplet beam about 1 mm downstream of the nozzle, we estimate that $\sim 5\times 10^6$ He droplets are present within the interaction volume given by the NIR beam focus. Therefore the number of doubly excited droplets available for nanoplasma ignition is still substantial despite the relatively low double-excitation probability.

In previous experiments carried out at an XUV free-electron laser (FEL), a higher XUV photon flux led to the excitation of pairs of He$$^*$$ with short interatomic distances $$\lesssim 1$$ nm; these pairs decayed by an ultrafast ($$\lesssim 1$$ ps) ICD process driven mainly by the quantum fluid dynamics of the He surrounding the He$$^*$$^19^. In the current HHG experiment, a similar signature of ICD is observed despite the much lower photon flux.

To unambiguously identify the presence of ICD under our current experimental conditions, we also measured photoelectron spectra from the droplets irradiated by the XUV pulse ($h\nu \approx 21.8$ eV). At this photon energy, He droplets are resonantly excited to their 1s2p $^1$P state which relaxes to the long-lived 1s2s $^1S$ state within $\lesssim 1$ ps^13,15^. Figure 3 shows the electron kinetic energy spectrum recorded for He droplets containing an average number of $5\times 10^5$ He atoms irradiated by only the XUV pump pulse. The peak at 15.55 eV arises from the emission of electrons created by ICD according to Eq. (1), confirming ICD being active under our experimental conditions. The peak is slightly shifted to lower energy with respect to the expected energy $E_e = 2\times E^* - E_i^\textrm{He}= 16.6$ eV, taking the atomic term value $E^*=20.6$ eV of the 1s2s $^1$S state into account. This shift and the asymmetric line shape are probably due to elastic scattering of ICD electrons at the He atoms in the droplets^44^.

In the present case of large droplets and low XUV photon flux, the average distance between the two interacting He$^*$ atoms is much larger as the density of He$^*$ is lower. Thus, ultrafast ICD mediated by the He quantum fluid dynamics is not expected to occur with high probability. Note that in most FEL experiments, in particular for large He nanodroplets^19^, the ICD electron yield at positive delays $\ge 3$ ps did not fully rise up to the level measured at negative delays. This indicated that another slower ICD channel is active in addition to the reported fast ICD process.

### Time-resolved XUV fluorescence emission

**Fig. 4:**
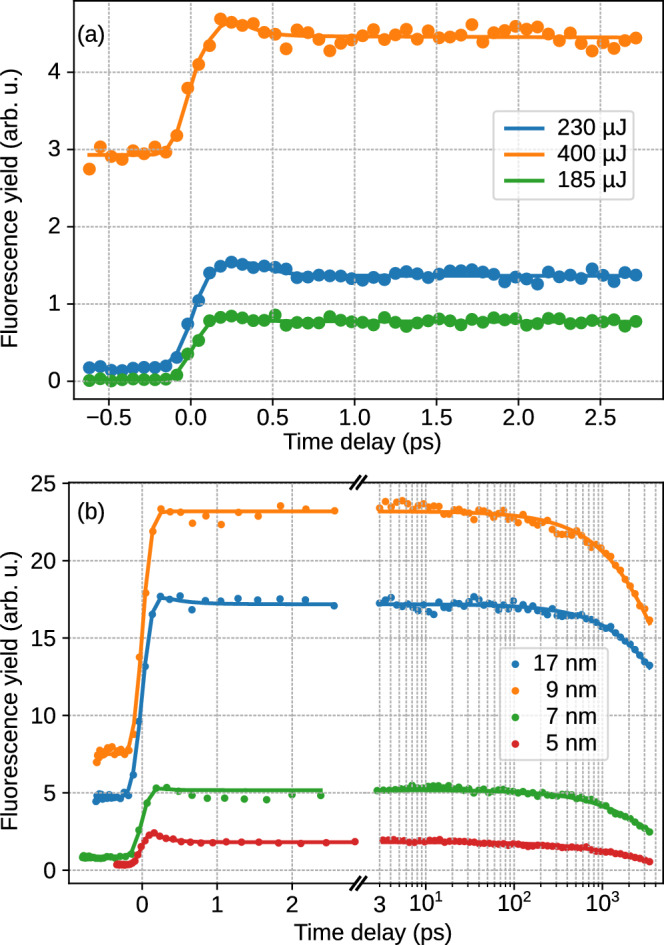
(**a**) Fluorescence yield as a function of pump-probe time delay for different NIR pulse energies for a He droplet with a radius of 7 nm containing 30,000 He atoms. For higher pulse energy, a nanoplasma is produced before the excitation of the droplet by the XUV pulse. (**b**) Fluorescence yield as a function of pump-probe time delay for different droplet sizes. Each curve shows two different decay time constants: A fast decay within $< 1$ ps and a slow decay on the ns scale. Each curve was fitted by a convolution of two exponential decay functions and the cross-correlation of pump and probe pulses given by Eq. (4).

Our fluorescence measurements indeed point to the existence of a much slower ICD process that depletes the He$^*$ population on the nanosecond timescale. Figure 4 shows the total fluorescence yield of He$^{+*}$ as a function of the delay between XUV pump and NIR probe pulses. The colored lines in Fig. 4a show total fluorescence yields of He$^{+*}$ for different NIR pulse energies for a He droplet of mean radius $R=7$ nm containing 30,000 atoms. At negative delay values, when the NIR probe pulse arrives before the XUV pump pulse, the NIR pulse is inefficient in causing tunnel ionization of He and igniting a nanoplasma when the NIR pulse energy is low ($\le 230~\mu$J). Thus, we measure only low yields of nanoplasma fluorescence. Only at a higher NIR pulse energy of 400 $\mu$J, corresponding to a peak intensity of $\lesssim 10^{15}$ Wcm$^{-2}$, does tunnel ionization occur in the largest He droplets present within the droplet size distribution. The ignition of the nanoplasma in large He droplets is facilitated by the higher probability for tunnel ionization of one or a few He atoms, as well as the tendency of large He droplets to pick up impurity molecules from the residual gas, mostly water, which serves as seeds for the NIR-driven avalanche ionization^45^. To systematically investigate the influence of the droplet size distribution on the ignition of nanoplasmas, we varied the droplet size by changing the nozzle temperature and recorded the delay-dependence of the integrated He$^{+*}$ fluorescence yield. A NIR probe pulse with a pulse energy of 230 $\mu$J was set to reach a high pump-probe signal contrast while maintaining a low level of nanoplasma ignition induced by the NIR pulse alone. Figure 4b shows the fluorescence yield as a function of delay for different average droplet sizes. Note the logarithmic scale at the abscissa. These delay-dependent fluorescence traces are characterized by

- Low fluorescence yields at negative pump-probe time delays.
- Increase of the fluorescence yield as the nozzle temperature is lowered to 12 K (mean droplet radius $R\approx 9$ nm).
- A decrease of fluorescence yield for nozzle temperatures below 12 K.
- A step-like rise in the fluorescence yield when the XUV pump and NIR probe pulses arrive simultaneously, and a peaking fluorescence yield around a delay of 200 fs.
- A marginal drop in fluorescence yield for pump-probe time delays between 0.2 ps and $\sim 1$ ps; this drop is more pronounced for smaller droplets.
- Most notably, an exponential decrease of the fluorescence yield on a long timescale (1-10 ns).

As the pump-probe time delay turns positive, the XUV pump pulse resonantly excites He prior to the NIR pulse. Although some fraction of the He$^*$ are likely to leave the droplets in the course of ultrafast droplet relaxation^13,18^, a large part of the He$^*$ remain bound to the droplet surface. These He$^*$ are tunnel ionized by the NIR pulse and thus act as seeds to drive the nanoplasma ignition in the same way as dopant atoms, due to their much lower ionization energy compared to that of ground-state He atoms. Therefore, we observe enhanced XUV fluorescence yields at positive delays. However, if the delay is long and the NIR pulse arrives after the He$^*$ have decayed or desorbed from the droplets, the nanoplasma ignition is again inefficient and the fluorescence yield drops.

As the nozzle temperature $T_0$ is lowered, the flux of He droplets out of the nozzle increases; however, the average size of He droplets also increases. Both a higher droplet flux and larger droplet sizes contribute to producing more excited and ionized He atoms, resulting in an overall rise in fluorescence yield. When lowering $T_0$ further into the super-critical expansion regime at $T_0\le 11$ K, where the mean droplet radius increases to $R\ge 17$ nm, the droplet number density drops and the droplet beam becomes more collimated. Under these conditions, the fluorescence yield decreases again. The fast depletion of He$^*$ by ICD mediated by the merging of adjacent bubbles forming around each He$^*$, which was discussed earlier^19^, is visible as a small drop in fluorescence yield between the peak of the signal at 0.2-0.3 ps and at about 1 ps delay. This fast dynamics is more pronounced for small droplets with $R=5$ nm, where the initial distance between two He$^*$ is short, mainly limited by the droplet size.

**Fig. 5:**
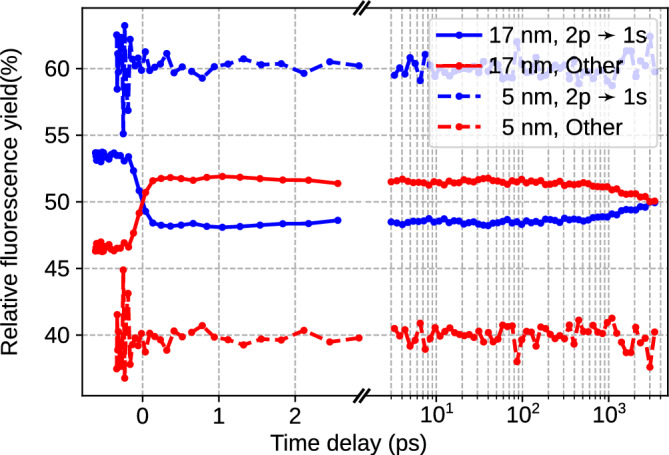
Relative fluorescence yield from the 2p state of excited He$^{+*}$ (blue) and all other states combined (red) as a function of pump-probe time delay for a NIR pulse energy of 230 $\mu$J. Solid lines show the relative yield from large droplets with a mean radius $R\approx 17$ nm, dotted lines are for small droplets with $R\approx 5$ nm.

The fluorescence yield from different excited states of He$^{+*}$ also shows a droplet size-dependent behavior. For a constant NIR pulse energy of 230 $\mu$J, the fluorescence from the 2p state decaying to the 1s state, compared to the fluorescence from higher excited states, is higher for small droplets and decreases for larger droplets. Figure 5 shows the relative fluorescence yields for smallest and largest droplet sizes in the experiment as a function of pump-probe time delay. Figures S2 and S3 in the Supplementary Material show additional systematic measurements of relative flurorescence yields for variable droplet sizes and different NIR pulse energies. The relative fluorescence from the 2p state is shown as blue symbols, while the sum of the relative yield of all higher states is shown in red. The solid lines show the relative yields for large droplets ($R\sim ~17$ nm), the dotted lines show the relative yields for small droplets ($R\sim ~5$ nm). It is well known that larger He droplets tend to be more efficiently avalanche ionized, thus forming a hotter and more highly charged nanoplasma^46^. The total amount of absorbed energy from the NIR probe, $W_{\text {abs}}$, is larger compared to small droplets. The same holds for optimum values of the pump-probe time delay, in the present case 0.2-1000 ps. Larger values of $W_{\text {abs}}$ lead to higher occupation of highly excited He$^{+*}$ states in the expanded nanoplasma. A more detailed analysis of the relative fluorescence yields at different experimental conditions is presented in the Supplementary Material.

To quantify the observed dynamics of the total fluorescence yield, the fluorescence yield curves were analyzed by fitting them to a model that takes into account an exponential drop at short delays ($<1$ ps) and at long delays ($\gtrsim 100$ ps). The model for the dynamics at short and long delays is given by

2 $$\begin{aligned} f_{s,l}(t) = \Theta (t) \exp \left( -\frac{t}{\tau _{s,l}}\right) , \end{aligned}$$

where $\Theta (t)$ is the Heaviside step function and $\tau _{s,l}$ are the decay time constants of the exponential functions that model the decays at short and long delays. Functions $f_{s,l}(t)$ were convolved with a Gaussian function that models the cross-correlation of pump and probe pulses. The resulting convolved functions are given by

3 $$\begin{aligned} \mathscr {F}_{s,l}(t) = \exp \left[ \frac{1}{2} \left( \frac{\delta }{\tau _{s,l}} \right) ^{2} - \frac{t}{\tau _{s,l}} \right] {\textbf {erfc}}\left[ \frac{1}{\sqrt{2}} \left( \frac{\delta }{\tau _{s,l}} - \frac{t}{\delta }\right) \right] . \end{aligned}$$

Here, $2\sqrt{2\ln {2}}\delta$ is the FWHM of the cross-correlation between pump-probe pulses. The final fit function is taken as the weighted sum of each convolved exponential decay function,

4 $$\begin{aligned} \mathscr {F}(t) = A \mathscr {F}_s(t) + B \mathscr {F}_l(t). \end{aligned}$$

The fit yields the value $\delta \approx 95 \pm 15$ fs corresponding to a cross-correlation FWHM between the pump and probe pulses of approximately 221 fs. This is similar to the expected value of 160 fs estimated from FWHMs of the XUV ($\sim$ 50 fs) and NIR pulses ($\sim$ 150 fs). The resulting value for the fast ICD process, $\tau _s \approx 200 \pm 100$ fs, is in agreement with those reported by Laforge et al.^19^. Values for the long decay constant $\tau _l$ inferred from the fit are compiled in Table 1. These values depend on the droplet size and range from about 2 ns up to $\sim 10$ ns for the largest droplets studied here.

**Table 1:** Comparison of the decay constants obtained from the experiment and from the Monte-Carlo molecular dynamics simulation.

Temperature (K)	Droplet radius (nm)	Decay constant (ns)	
		Experimental	Simulated
14.5	5	1.9	1.96
14.0	7	3.7	3.30
12.0	9	5.5	5.35
**11.0**	**17**	**8.6**	**15.15**

The long time constant $\tau _l$ takes values of a few nanoseconds, and it increases with the droplet size. Following the excitation of the droplet to the 1s2p $^1$P state, it relaxes to a state correlating to the 1s2s $^1$S state^13,15^. Subsequently, within $\sim 1$ ps, a void bubble forms around the excited He$^*$ atom on which the excitation localizes. The bubbles forming around adjacent He$^*$ either merge into one, leading to fast ICD^19^, or they are expelled to the droplet surface^13^. When the bubbles burst at the droplet surface, the released He$^*$ are either ejected away from the droplet^18^ or, for larger droplets, remain bound to the droplet surface^25^.

Due to the superfluid nature of He nanodroplets, embedded atoms and molecules have previously been found to freely move through the droplets^47–49^. Therefore, we assume that surface-bound He$^*$ roam on the He droplet surface freely without experiencing any friction as long as their velocity stays below a critical value, analogously to the frictionless motion in the droplet interior^49^. This free motion is interrupted only when two He$^*$ atoms approach each other closely enough for their orbitals to overlap, at which point decay via ICD occurs. Therefore, the ICD time constant is determined by the roaming motion of pairs of He$^*$ atoms at the droplet surface. The depletion of the population of He$^*$ by ICD consequently leads to quenching of the fluorescence emission.

### Numerical simulations

**Fig. 6:**
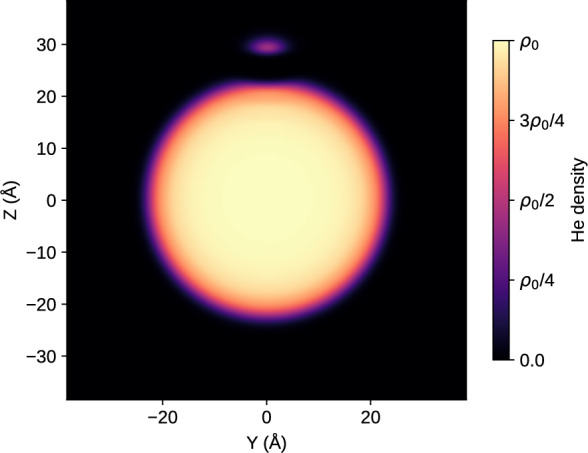
Helium density functional theory (DFT) simulation showing the probability density of the He$^*$ wave function localized at the surface of a He nanodroplet of radius $R=2.22$ nm consisting of $10^3$ He atoms. The He$^*$ atom is bound to the droplet surface with a binding energy of 9.7 K, and its center of mass is located at 2.95 nm from the droplet center. The color bar shows the He density in units of $\rho _0$, where $\rho _0$ is the density of bulk liquid He at zero temperature and pressure.

To assess our conjecture of He$^*$ roaming freely about the droplet surface and decaying by ICD upon their encounter, we performed a set of numerical simulations. First, we explored the equilibrium structure of a He$^*$ atom bound to a He nanodroplet. To this end, a static $^4$He density functional theory ($^4$He-DFT) simulation was performed for a pure He droplet containing 1000 He atoms ($R=2.22$ nm) supporting one He$^*$ atom on its surface. The He$^*$ was treated as a quantum impurity due to its low mass. As explained in Ref. 50, where details about the method can be found, $^4$He-DFT has proven to be the best compromise between accuracy and the ability to treat a large number of atoms. The He$^*$ in the 1s2s $^1$S state was described by a wave function and the rest of the droplet by its He density, both expanded on the same 3-dimensional Cartesian grid. The equilibrium configuration was found by solving the coupled Euler-Lagrange equations arising from functional variation of the equation expressing the total energy as a functional of the He density. The He$^*$-droplet interaction was taken as a sum of pairwise He$^*$-He potentials using the He$^*$(1s2s $^1$S)-He form of Fiedler and Eloranta^51^.

The simulations were performed using the free and open-source 4He-DFT-BCN-TLS code^52^ with the Orsay-Trento functional^53^. The resulting equilibrium configuration for the He$^*$(1s2s $^1$S)@He$_{1000}$ system is represented in Fig. 6 as a 2-dimensional cut in the polar plane. He$^*$ is bound to the droplet surface $\sim 2.95$ nm away from the center of mass of the droplet with a binding energy of $\sim 9.7$ K (0.84 meV). In response, the droplet surface forms a shallow dimple underneath the He$^*$. Thus, the simulation confirms that a He$^*$ atom can be stably bound to the surface of a He droplet.

**Fig. 7:**
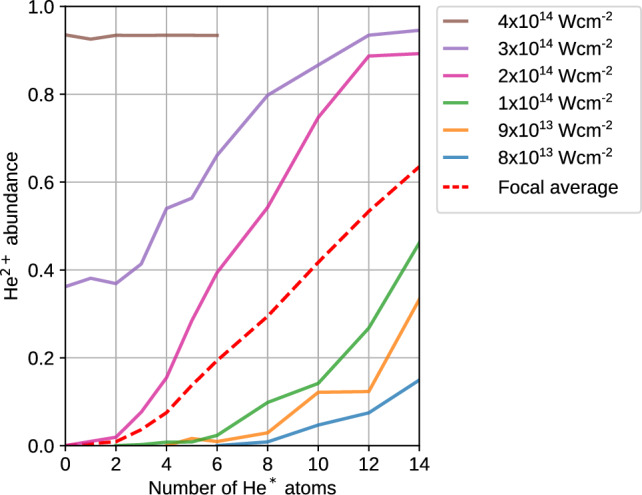
MD simulation of the He$^{2+}$ ion abundance as a function of the number of excited He$^*$ atoms attached to a He nanodroplet when the NIR pulse arrives. Different colors represent different intensities of the NIR pulse. The dashed red line represents the focal average over NIR intensities from $8\times 10^{13}$ to $2\times 10^{14}$ Wcm$^{-2}$. From the focal averaged He$^{2+}$ abundance, it is clear that only a few He$^*$ atoms are needed as seeds to ignite a nanoplasma in the droplet.

Second, to assess the He$^*$ capacity to facilitate the ignition of a nanoplasma in a He nanodroplet, we performed classical molecular dynamics (MD) simulations similar to those in earlier works^28,40,54^. The He$^*$ were modeled by assigning them the excitation energy of 20.6 eV of the 1s2s $^1$S state, lowering their ionization energy by the corresponding amount. These simulations were initialized by randomly placing a small number $n^*$ of He$^*$ inside a He nanodroplet containing 2171 He atoms ($R=2.6$ nm). The evolution of this system under irradiation by an NIR pulse of 100 fs duration and various pulse peak intensities was computed until 220 fs after the NIR pulse maximum, when the ionization avalanche converged. For each $n^*$ and pulse peak intensity, a set of 100 trajectories with different random positions of the He$^*$ was simulated and the abundance of He$^{2+}$ dications, which are the precursors of the experimentally observed nanoplasma fluorescence, was averaged over the trajectory set. Figure 7 shows the He$^{2+}$ trajectory-set abundances as a function of $n^*\le 14$ for various pulse peak intensities (solid curves). Depending on the NIR pulse intensity, either $\gtrsim 6$ He$^*$ ($I=10^{14}$ Wcm$^{-2}$) or $\gtrsim 2$ He$^*$ ($I>2\times 10^{14}$ Wcm$^{-2}$) are needed to trigger ignition of a nanoplasma. At intensities $I\ge 3\times 10^{14}$ Wcm$^{-2}$, even pure He nanodroplets that do not contain any He$^*$ are efficiently avalanche ionized.

For a better comparison with the experimental data, the dashed red curve shows the He$^{2+}$ ion abundance averaged over various intensities according to the intensity distribution in the volume determined by the spatial overlap of the XUV and the NIR beams. The focal averaging involves the intensities that contribute to the He$^{2+}$ abundance, up to $I\le 2\times 10^{14}$ Wcm$^{-2}$. For this intensity distribution, the probability of avalanche ionization of pure He nanodroplets containing no He$^*$ excitations is negligible as in the experiment for NIR pulse energies $<230~\mu$J (Fig. 4a). Clearly, at NIR pulse intensities close to the threshold for avalanche ionization of the pure He nanodroplet ($\sim 3\times 10^{14}$ Wcm$^{-2}$), exciting just a few He$^*$ already leads to nanoplasma ignition in some trajectories. This confirms that resonant excitation is another efficient route to activation of He nanodroplets for subsequent NIR-induced nanoplasma ignition, similar to photoionization of the nanodroplets that led to the formation of stable He$^+_n$ ‘snowball complexes’^28^. However, in contrast to those earlier experiments, in which the probability of nanoplasma ignition remained constant over many nanoseconds, the activation by resonant excitation decays on the nanosecond timescale. The focally averaged He$^+$ abundance shown in Fig. S4 is by a factor of 6 smaller than that of He$^{2+}$. The weak droplet size dependence of the ignition probility, see Fig. S5, suggests that the simulation results for He nanodroplets containing 2171 He atoms are applicable to larger droplet sizes as well.

The MD simulations also show a direct correlation between $W_{\text {abs}}$ and the radius of the classical Kepler orbit $\langle a \rangle$ of an electron around a He$^{2+}$ ion, see Fig. S6. This is in line with the experimental results which show that ignition of larger droplets leads to the occupation of higher excited He$^{+*}$ states, see Fig. 5. Details of the MD simulation method including the focal averaging procedure and the simulation results are presented in the Supplemental Material.

**Fig. 8:**
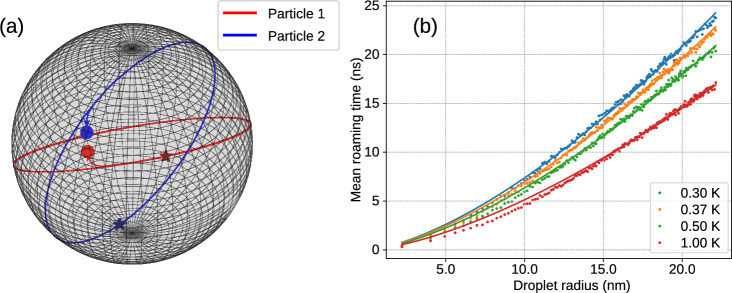
(**a**) Illustration of two excited He$^*$ atoms roaming on the surface of a He droplet as simulated by a classical trajectory calculation. Atoms 1 and 2 are initialized at random positions marked by the symbols $\star$ and velocities randomly generated from a Maxwell-Boltzmann distribution at a temperature of 0.37 K; we neglect the weaker He-He$^*$ interaction and retain only the stronger He$^*$-He$^*$ interactions. The colored symbols $\bullet$ indicate the endpoints of the trajectories. Both particles start their trajectories with constant velocities along the droplet’s surface, following circular orbits on the sphere until they reach a short interatomic distance where they mutually attract each other and subsequently decay via ICD. (**b**) Mean roaming times of He$^*$ atoms before they meet and decay by ICD as a function of droplet radius; each value is the result of averaging over $10^5$ Monte-Carlo trajectories. Different colors show different initial velocity distributions given by the indicated temperatures. The simulated curves are fitted to a simple power-law, and in the case of $T=0.37$ K, the best fit is deduced so that the time constant scales as $\tau = 0.21R^{3/2}$.

To rationalize the long decay dynamics observed in the experiment, in a third step, we modeled the motion of He$^*$ atoms along the surface of a nanodroplet, assuming that two colliding He$^*$ decay by ICD. To this end, classical trajectories of two He$^*$ were simulated using the Monte Carlo method; the initial positions of the atoms were chosen randomly from a 3D uniform distribution of coordinates on the surface of the sphere. The magnitude of the initial velocity was randomly generated according to a Maxwell-Boltzmann distribution for the He droplet temperature of 0.37 K. The initial velocity vector of each He$^*$ was randomly oriented in a plane tangential to the surface at its initial position. Previous experiments, in which the temperature of an atom or molecule attached to a He nanodroplet was measured, showed that the dopant atom or molecule fully thermalized to the droplet temperature $T=0.37$ K^55,56^. Therefore, it appears probable that also the translational degree of freedom of a particle attached to a He nanodroplet thermalizes to the nanodroplet temperature^30^. Another plausible value would be the critical Landau velocity for frictionless motion of a molecule through superfluid He^49,57^. It turns out that for He$^*$, the thermal velocity, $v_\textrm{th} = \sqrt{8 k_\textrm{B} T / (\pi m_\textrm{He})} = 44$ m/s has a similar value at $T = 0.37$ K as the critical Landau velocity, $v_c \approx 60$ m/s. Using the velocity Verlet integrator^58^, Newton’s equation of motion was solved numerically for each atom, taking into account only the attractive force acting between the He$^*$ resulting from to the He$^*$-He$^*$ pair potential for 1s2s $^1S$ states^51^, and neglecting the interaction between the He$^*$ and the droplet. The code *Sphere roaming* is available under an open-source license^59^.

Figure 8a illustrates one sample trajectory of a pair of He$^*$ atoms orbiting around the sphere representing the He nanodroplet. The randomly chosen initial positions of the atoms are marked with the symbols $\star$ and the final positions are marked with $\bullet$. The attractive force acting between the two He$^*$ has almost no effect as long as the distance between the He$^*$ exceeds $\sim 3$ nm. Therefore, unless both particles were initialized at a distance $\le 3$ nm, they follow circular orbits around the droplet, deviating from these orbits only when coming close to each other. When the atoms approach each other to less than 0.6 nm, the simulation is stopped, and the time it took the atoms to collide was recorded. For each droplet size $10^5$ trajectories were simulated.

The resulting mean roaming time as a function of droplet radius *R* is shown in Fig. 8b. Remarkably, at $T=0.37$ K, the mean roaming time follows a simple power law $\tau \propto R^{3/2}$. This scaling lies between $\propto R$, which would be expected for direct collisions between He$^*$ roaming along the identical one-dimensional closed orbit, and $\tau \propto R^2$, which would be expected for diffusive motion of the He$^*$ exploring the entire surface area of the droplet. Our simulation of He$^*$ moving along closed circular orbits, which, however, are randomly oriented with respect to each other and each He$^*$ has a different velocity according to the thermal distribution, can be viewed as an intermediate case between the two scenarios described above. Therefore, the observed $\tau \propto R^{3/2}$-scaling appears highly plausible.

Assuming only two He excitations occur in each droplet, the roaming time was calculated as an average over the log-normal droplet size distribution for a given mean droplet size sampled with $10^7$ values. The resulting time constants $\tau _l$ match the experimental observations except for the case of large droplets with a mean radius $R=17$ nm, see Table 1. A potential source of disparity between the experimental and simulated values lies in the determination of droplet sizes, particularly for larger droplets, as the average droplet size was inferred from the literature rather than directly measured. Moreover, larger droplets have a finite probability of accommodating more than two He$^*$ per droplet, which reduces the roaming time.

### Alternative processes

While we take the good agreement between the results of our experiment and simulations as a confirmation of our interpretation in terms of ICD of He$^*$ roaming on the surface of He nanodroplets, possible alternative interpretations should be discussed.

*Desorption of He*$^*$* from droplets;* The first time-resolved experiments on Rydberg-excited He nanodroplets seemed to indicate that He$^*$ excited atoms are readily ejected from the droplets^18^. Ejection or desorption of He$^*$ from the droplets would quench the ICD process. However, subsequent measurements showed that the ejection of He$^*$ atoms is actually a minor relaxation channel^60,61^. This finding is supported by another observation: Foreign atoms or molecules attached to the He droplets (‘dopants’) are efficiently ionized by ICD through interaction with He$^*$, a process traditionally termed Penning ionization^12,62,63^. Thus, we assume that He$^*$ atoms predominantly remain bound to the droplet surface over periods longer than typical ICD decay times.

*He*$_2^*$* excimer formation and fluorescence decay;* Upon optical excitation, He$^*$ is formed in a singlet state which predominantly relaxes to the long-lived 1s2s $^1$S state irrespective of the initial excited state^13–15,25^. Direct fluorescence decay from the initially excited 1s2p $^1$P state back to the ground state is only a weak channel^64^. In bulk liquid He, He$_2^*$ excimers form in the $A\,^1\Sigma _u^+$ state by association of the He$^*$($^1$S) with a neighboring ground-state He atom despite the presence of a repulsive potential energy barrier at intermediate distance, likely due to many-body interactions^65^. When He$_2^*$ excimers are directly formed in their vibrational ground state, they decay by fluorescence emission within $<10$ ns^66^ which comes close to $\tau _l$ measured in the present experiment for large droplets. This excimer fluorescence may underlie the ‘short-lived’ XUV fluorescence (2.5-7.5 ns after excitation) previously detected from resonantly excited He nanodroplets using synchrotron radiation^67^. However, in He nanodroplets, only a very small fraction of He$^*$ atoms form He$_2^*$ excimers after resonant excitation, as found in simulations and in earlier time-resolved experiments^61,68^. In particular, neither photoionization electron spectra^13,15^ nor ICD electron spectra^25,26,62^ have shown any significant contribution from He$_2^*$ excimers formed in He nanodroplets in this regime of photoexcitation. In He nanodroplets, ICD involving He$_2^*$ excimers is observed only in the regimes of autoionization and electron-impact excitation at $h\nu >23$ eV and $h\nu >45$ eV, respectively^12,26,69^. This leads us to conclude that in the low-density environment given at the surface of He nanodroplets, the repulsive potential barrier in the He$^*$-He pair potential^51,70^ efficiently prevents the formation of He$_2^*$ excimers; therefore, we do not expect He$_2^*$ excimers and their fluorescence decay to play a role in the current experiment. Non-radiative decay of He$^*$ by internal conversion can be safely ruled out as the potential energy curves of He$^*$He pairs are far separated from the He$_2$ ground state potential. In contrast, ICD is clearly active under the present experimental conditions, and depletion of the He$^*$ population by ICD appears to be the main process responsible for the observed decay dynamics.

*Exciton-exciton annihilation*; A similar but distinct process to ICD is exciton-exciton annihilation (EEA) occurring in different classes of nanoscale and extended condensed-phase systems, such as molecular aggregates and crystals, as well as certain photosynthetic pigments. At high excitation densities, EEA proceeds by fusion of two excitons. In this process, energy transfer takes place between the two excitons, leading to the annihilation of one exciton^71^. EEA is driven by purely electronic energy transfer and does not involve the mechanical motion of the molecules themselves, except for local vibrations that provides the energy match needed for resonance between donor and acceptor sites. It can occur by transfer of electronic excitation energy from one excited molecule to another by direct Förster transfer (via resonant dipole–dipole coupling) or Dexter transfer (via orbital overlap, corresponding to the ICD discussed here); in diffusive or multistep transfer, energy is transferred to a unexcited molecule followed by exciton annihilation^72^. At high excitation densities, direct EEA initially dominates. As the excitation density decreases and the average distance between excitons increases, diffusive EEA predominates. These exciton transfer mechanisms, while similar to the ICD process, involve two transitions between discrete energy levels and thus require resonance conditions for energy conservation. In contrast, in ICD the accepting molecule is ionized with the emitted electron carrying away the excess energy, thus offering more flexibility in conserving energy. Low photon fluence and large droplet size reduce the He$^*$ concentration, thus favoring the roaming ICD mechanism that resembles diffusive (Förster or Dexter) exciton transfer. However, diffusive transfer occurs via exciton hopping within a static molecular assembly, whereas the analogous process in He droplets relies on the ballistic motion of excited He$^*$ atoms over large distances, enabled by the superfluid nature of He nanodroplets.

## Conclusion

In summary, we studied the evolution of resonantly excited He nanodroplets using XUV fluorescence as a novel probe for the dynamics occurring on various time scales. Our experiment, backed by a set of simulations, demonstrates that resonant excitation of He nanodroplets by an XUV pulse efficiently activates the droplets for subsequent avalanche ionization and nanoplasma formation by a subsequent NIR pulse, even for a small number of excitations per droplet. The most abundant XUV fluorescence emission from the nanoplasma was measured for transitions from the lowest excited levels to the ground state of the He$^+$ ion. The fluorescence yields showed a slight drop at pump-probe time delays between 0.2 ps and $\sim 1$ ps, reflecting the fast decay of excited states by ICD mediated by the merging of bubbles formed around He$^*$ excitations. More prominently, the fluorescence yield slowly drops on a time scale of several nanoseconds. This slow dynamics is rationalized by the decay of pairs of droplet-bound excited He atoms by ICD, based on the detection of characteristic electrons and backed by numerical simulations. Given the low concentration of He$^*$ excitations in each nanodroplet, the He$^*$ emerge to the nanodroplet surface, orbit around the nanodroplet, and eventually decay by ICD upon their encounter. This process can be seen as an extreme case of ICD driven by nuclear motion, in contrast to most other previously reported instances of ICD where pure electronic couplings have been the main driving mechanism^1^.

Further experiments are planned that will resolve pump-probe delay-dependent ICD electron spectra, both for the given scenario of resonant excitation and for other ICD schemes such as He droplet excitation by photoelectron impact^69^, photo-double excitation^73^, and simultaneous photoionization and excitation^74^. Bright and tunable laser-based high-harmonic sources, which are now becoming readily available^32^, are particularly well suited for such experiments as clusters and nanoparticles have large total absorption cross sections in the XUV range owing to their sheer size; this allows us to study the ultrafast dynamics of collective excitations^19,32^ and multiple photoionization processes^28^ with excellent time resolution, revealing complex interactions of nanoscale matter with ionizing radiation that may have implications for a range of related fields, including radiochemistry and radiation biology.

## Supplementary Information

Supplementary Information.

## Data Availability

The datasets used and analyzed during the current study are available from the corresponding author upon reasonable request.
