# Supplementary material for: XUV fluorescence as a probe of interatomic coulombic decay of resonantly excited He nanodroplets
Source: Sci Rep. 2026 Jan 9;16:1321. doi: 10.1038/s41598-025-34677-x (PMC12796158; doi:10.1038/s41598-025-34677-x)
Supplement: Supplementary file 1 — Supplementary Information. [file 41598_2025_34677_MOESM1_ESM.pdf]

Supplemental Material: XUV fluorescence as a probe of interatomic Coulombic decay of resonantly excited He nanodroplets

Keshav Sishodia *et al.*

1 Excitation probability

Figure S1 shows the excitation probability for single and multiple excitation of a He nanodroplet for a given mean droplet radius $\langle R \rangle$. For droplets containing a mean number of He atoms $\langle N \rangle$, the average number of excitations is

$$\lambda = \Phi\sigma\langle N \rangle, \tag{1}$$

where Φ is the photon fluence of one XUV pulse and $\sigma \approx 15 \times 10^{-22}$ cm^{-2} is the absorption cross-section of one He atom in a droplet at $h\nu = 21.8$ eV. $\langle N \rangle$ and $\langle R \rangle$ are related by

$$\langle N \rangle \approx \frac{4}{3}\pi \langle R \rangle^3 \rho_{\mathrm{He}}, \tag{2}$$

where $\rho_{\mathrm{He}} \approx 21.8$ nm^{-3} is the He number density in He nanodroplets.

The probability of k excitations for a given droplet size is given by the Possionian distribution

$$P_k = \frac{\lambda^k e^{-\lambda}}{k!}. \tag{3}$$

Thus, for $\langle N \rangle = 7 \times 10^4$ ($\langle R \rangle = 9$ nm), the probability of zero, one, two and more that two excitations per droplet are $P_0 = 84\,\%$, $P_1 = 14.6\,\%$, $P_2 = 1.3\,\%$, $P_{k>2} = 0.1\,\%$.

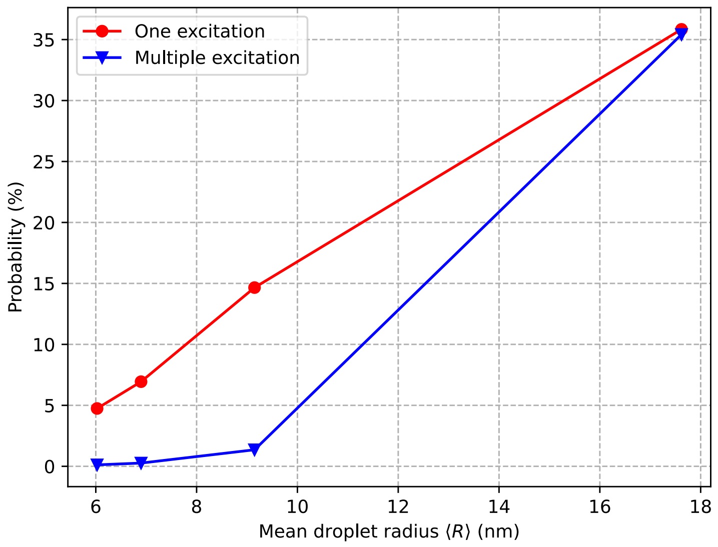

Figure S1: Probability of single and multiple (≥ 2) excitation of He nanodroplets as a function of mean droplet radius $\langle R \rangle$ according to the Possionian distribution.

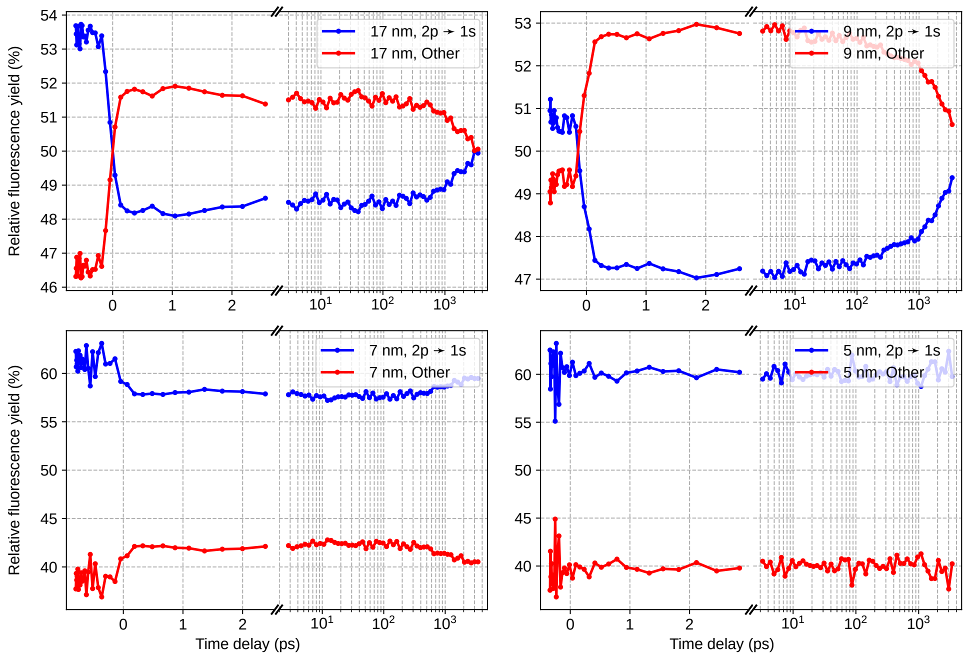

Figure S2: Relative fluorescence yield as a function of pump-probe time delay. The blue line indicates the relative fluorescence yield from the 2p→1s state relative to the total fluorescence yield, while the red line shows the yield from all higher states. At negative delays, fluorescence arises from nanoplasma created solely by the NIR pulse. At positive delays, nanoplasma is generated by the tunnel ionization of excited He^* atoms by the NIR pulse. For larger droplets, the fluorescence from higher states is stronger than that from the lowest fluorescing 2p state.

2 State-resolved fluorescence yields

Figure S2 shows the relative fluorescence yield for different droplet sizes as a function of the pump-probe time delay. Relative fluorescence yield from the 2p state of excited He^{+*} is shown in blue color, while the relative yield of all the other states combined is shown in red color. At negative delays, fluorescence arises from nanoplasma created by inefficient ignition of the droplet solely by the NIR pulse, which shows a higher fluorescence yield from the 2p→1s state. For large droplets, the fluorescence from higher states dominates over the fluorescence from the 2p→1s state.

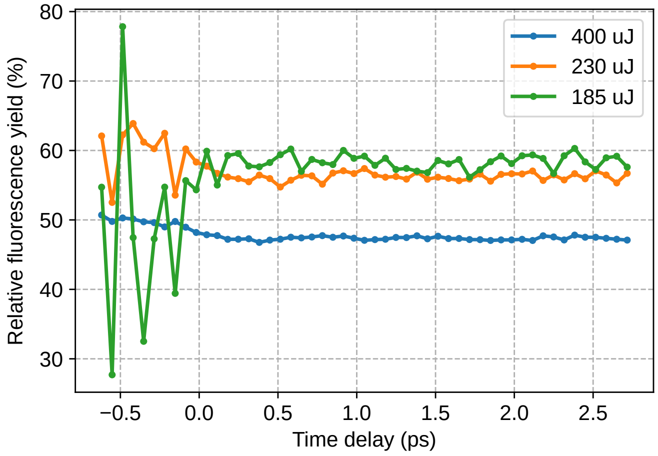

Figure S3: Relative fluorescence yield of the 2p state to the total fluorescence yield as a function of pump-probe delay for different NIR pulse energies for He droplets with mean radius of 7 nm containing 3×10^4 He atoms. For high NIR pulse energies, the relative fluorescence yield from the 2p state drops, thereby increasing the relative fluorescence yield from higher states.

Figure S3 shows the fluorescence yield from the lowest fluorescing 2p state relative to the total fluorescence for different NIR pulse energies. For higher pulse energies, the He droplet absorbs more energy, accumulating more quasifree electrons and ions. This, in turn, leads to a higher ion-electron recombination rate, leading to the occupation of higher-lying states of He^{+*}, thus increasing the relative fluorescence yield from higher states and decreasing that of the 2p→1s transition. See figure 4 (a) in the main text for the total fluorescence yield as a function of pump-probe time delay.

3 Molecular dynamics simulations

The goal of the molecular dynamics (MD) simulations in this work is to explore how many electronically excited He atoms have to be generated in a droplet by the XUV pump pulse so that the NIR probe pulse triggers ("ignites") an ionization avalanche, as well as to simulate the produced He^+, He^{2+} ion yields.

The general features of the MD simulation method for the interaction of a

cluster with the electric and magnetic field of a linearly polarized NIR Gaussian laser pulse was described earlier.[1–4] In short, all nuclei and nanoplasma electrons are treated classically, starting with a cluster of neutral atoms. Electrons enter the MD simulation when the criteria for tunnel ionization (TI), classical barrier suppression ionization (BSI) or electron impact ionization (EII) are met. The criteria for TI, BSI and EII are checked at each atom at every MD time step, using the local electric field at the atoms as the sum of the laser electric field and the contributions from all ions and electrons of the cluster. Instantaneous TI probabilities are calculated by the Ammosov-Delone-Krainov formula,[4,5] and EII cross sections by the Lotz formula,[6] taking the ionization energy with respect to the atomic Coulomb barrier in the cluster.[7] Interactions between ions are described by Coulomb potentials, electron-ion and electron-electron interactions by smoothed Coulomb potentials. Interactions involving neutral atoms are disregarded except for a Pauli repulsive potential between electrons and neutral He atoms. Accordingly, the current simulation model cannot account for excited state nuclear dynamics which would require the incorporation of at least He^*-He as well as He-He pairwise potentials. Only from the instant of the first ionization on, the simulation model can describe nuclear and electron dynamics.

The role of the electronic excitation is to activate the droplet for the subsequent ionization avalanche by the NIR probe pulse. The activation consists of reducing the effective ionization energy from 24.6 eV for ground state He by the electronic excitation energy to 4 eV for excited state He^*. While in the experiment He is excited into the 1s2p ^{1}P state and quickly relaxes to the 1s2s ^{1}S state with $\lesssim 0.5$ ps,[8,9] in our simulations we directly prepare the He^* atoms in their 1s2s ^{1}S state, corresponding to the excitation energy of 20.6 eV. Simulations are carried out for a fixed number n^* of He^* atoms, n^* being a fixed given value for a trajectory. Since excited state dynamics cannot take place in our simulation model and n^* is a predetermined value, the XUV pulse parameters are irrelevant as long as the electronic excitation is sufficiently separated from the NIR probe pulse in time.

We prepare the n^* He^* atoms randomly distributed over the droplet 300 fs prior to the NIR pulse maximum, which is sufficiently long given the intensity FWHM of the NIR probe pulse of 150 fs. For each value of n^*, a set of 100-200 trajectories is simulated, each with different random sites of the He^* atoms. We restrict our simulations to the abundances of bare ion charges He^+ and He^{2+}, that is three-body recombination (TBR) which is the necessary prerequisite for the experimentally observed fluorescence via the formation of

excited-state ions like the hydrogen-like He^{+*} ion, is not considered. Thus, by simulating bare ion charge abundances we obtain an indirect measure for the ability of the nanoplasma to fluoresce, assuming that the bare ion yield and the fluorescence yield are proportional to each other. Classical TBR (involving two electrons and the target ion) occurs automatically in classical trajectory simulations but would require long trajectories for letting the nanoplasma expand and cool. Since the goal of these simulations is to merely confirm the experimental observation that weak resonant excitation of He nanodroplets leads to their activation for subsequent nanoplasma ignition, we refrain from a more thorough modeling of the long-term dynamics that determines their fluorescence emission. The trajectories are propagated until 220 fs after the NIR pulse maximum at which point the ionization avalanche has fully developed.

For a better comparison with the experiment, the simulation results are averaged over the intensity profile in the NIR focal spot. A corresponding focal averaging over the XUV intensity profile is not necessary, since the trajectories are run for fixed given values of n^*. For the focal averaging of the NIR focal spot we start from a 3D intensity profile of a Gaussian beam[10]

$$I(r,z) = I_{\max}\frac{w_0^2}{w(z)^2}\exp\left(-\frac{2r^2}{w(z)^2}\right), \tag{4}$$

where $I_{\max}$ is the intensity in the center of the focal spot, $w(z) = w_0\sqrt{1+z^2/z_0^2}$ is the z-dependent beam radius, w_0 is the beam radius in the beam waist, z is the propagation direction of the photons and r the radial coordinate perpendicular to z. For the Rayleigh length $z_0 = \pi w_0^2/\lambda$ the ratio is $z_0^{\mathrm{XUV}}/z_0^{\mathrm{NIR}} = 54$, taking the focal spot size w_0^2 of the XUV ($\lambda = 57$ nm) to be 64×47 $\mu\mathrm{m}^2$ and the focal spot size of the NIR ($\lambda = 796$ nm) to be 18×43 $\mu\mathrm{m}^2$. Thus, the XUV intensity profile is considerably more elongated than that of the NIR beam. Since in the experiments both beams are aligned nearly collinearly, the XUV intensity profile cuts through the 3D NIR volume along the z-axis and activates only droplets along a narrow channel. Accordingly, focal averaging reduces approximately to a 1D averaging over a Lorentzian intensity profile along the z-axis,

$$I(z) = I_{\max}\frac{z_0^2}{z_0^2+z^2}. \tag{5}$$

The 1D focal averaging is carried out analogously to the procedures for 2D and 3D intensity profiles described in Ref.[11]. To this end, MD simulations

are carried out for selected NIR pulse peak intensities between 8×10^{13} and 2×10^{14} Wcm^{-2}, i.e., from the lowest intensity at which droplets contribute to the ion yield up to the highest sampled intensity at which no ignition occurs in absence of He*.

The simulations are carried out mainly for the He_{2171} droplet, whereas the smallest average droplet size considered in the experiment is 10^4 He atoms. Some simulations are also performed for droplet sizes up to 10^4 atoms to discuss the droplet size dependence of the ion signals. The structures of the droplets is assumed to be a FCC lattices with a He-He distance of 3.6 Å.[12]

As stated in the main text, the focally averaged simulated He^+ abundance (normalized to the total number of He atoms in the droplet) is by a factor of six lower than that of He^{2+}. Figure S4 exhibits the He^+ and He^{2+} abundances. Presented are the focally averaged results (green curves) as well as the results for the lowest and highest single NIR pulse peak intensity involved in the focal averaging. At the lowest intensity, $I = 8 \times 10^{13}$ Wcm^{-2} (blue curves), the He^+ and He^{2+} abundances are comparable, while at the highest intensity, $I = 2 \times 10^{14}$ Wcm^{-2} (magenta curves), He^{2+} predominates by far. As a general trend, for a fixed intensity the abundances of both He^+ and He^{2+} increase with increasing n^*, because ignition takes place in more trajectories. Further, the presence of more He*s tends to ignite the droplet at earlier times during the NIR pulse, leading to a more efficient avalanche and thus the generation of more He^{2+} per single trajectory.

Figure S5 shows the droplet size dependence of the ignition probability for five droplet sizes up to $\approx 10^4$ atoms. The ignition probability is defined as the fraction of trajectories of a set in which an ionization avalanche is observed. As an ignition criterion we take a minimum threshold of 200 ionizations in the droplet. The threshold value is uncritical as the ionization avalanche was found to propagate through all the droplet once the threshold is exceeded. At $I = 3 \times 10^{14}$ Wcm^{-2} and without the presence of He*s (red curve), a strong droplet size dependence is exhibited, as the cumulative tunnel ionization probability, which grows with the number of atoms in the droplet, reaches a level at which a single tunnel ionization somewhere inside the droplet occurs, is sufficient at this intensity to trigger an avalanche. While for the He_{2171} droplet, for which the simulation results of Figure 7 in the main text are carried out, the ignition probability amounts to only 0.35, for the average droplet size range $\geq 10^4$ atoms ignition occurs with near certainty. For $I = 2 \times 10^{14}$ Wcm^{-2}, ignition probabilities are presented for $n^* = 4$ and 6. After a steep rise with a weak maximum at 2×10^3 atoms, the

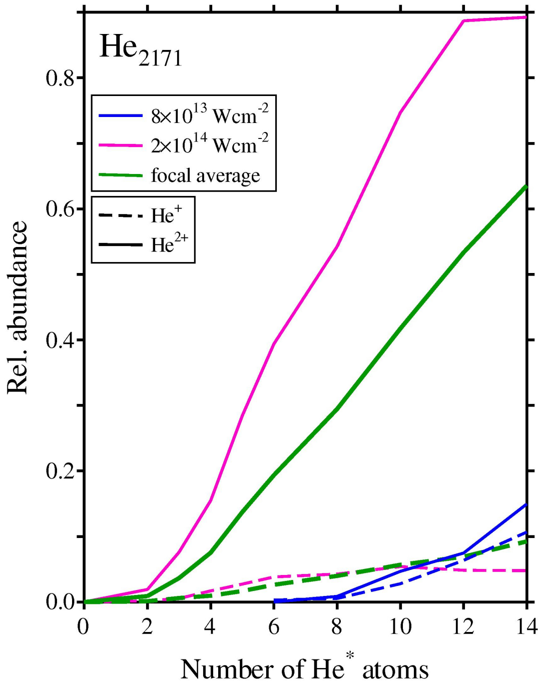

Figure S4: He^+ (dashed curves) and He^{2+} (solid curves) abundances for the He_{2171} droplet as a function of the number of He^* atoms. The green curves represent the focally averaged results involving all intensities $8 \times 10^{13} \leq I \leq 2 \times 10^{14}$ Wcm^{-2} presented in figure 7 of the main text. Given are also the results for the single intensities $I = 8 \times 10^{13}$ (blue) and $I = 2 \times 10^{14}$ Wcm^{-2} (magenta).

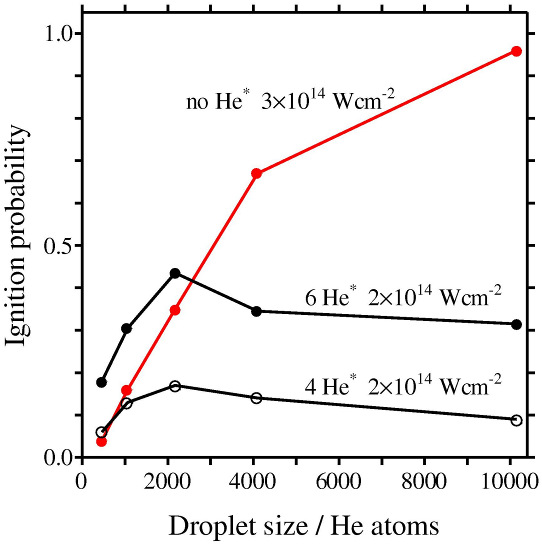

Figure S5: The droplet size dependence of the ignition probability, shown for the droplet sizes of 459, 1016, 2171, 4096, and 10149 He atoms. Given are three examples: $I = 3 \times 10^{14}$ Wcm^{-2}, $n^* = 0$ (red curve); and $I = 2 \times 10^{14}$ Wcm^{-2}, $n^* = 4$ and 6 (black curves).

probabilities show a weak droplet size dependence, only slightly decreasing towards larger droplet sizes. The cause for the weak maximum at 2×10^3 atoms is unknown. One may speculate about two opposite trends which may result in a maximum for the ignition probability: (1) With increasing droplet size, the electrons released from the He*s have a longer path through the droplet and consequently have more opportunities to cause secondary ionizations. (2) With increasing droplet size the probability decreases that two He*s are generated in a close neighborhood in the droplet. The He^+ ions generated from the He*s and by secondary ionizations then have fewer opportunities to collectively lower the Coulomb barriers at neutral He atoms in a specific region in the droplet.

The weak droplet size dependence of the ignition probability at $I = 2 \times 10^{14}$ Wcm^{-2} suggests that the simulation results for the He_{2171} droplet are applicable to the much larger droplets in the experiment. Further and not shown in figure S5, the absence of tunnel ionization for $n^* = 0$ even for droplets of 10^4 atoms extends the validity of 2×10^{14} Wcm^{-2} as the maximum intensity for the focal averaging at least up to this droplet size.

Figure S6 shows a characterization of the classical orbits of He^{2+}-electron system formed by three-body-recombination in the course of MD trajectories of He_{2171} droplets. Shown are the average semimajor axis values $\langle a \rangle$ of the electrons in their elliptical Kepler orbits around the central He^{2+} ions. Every data point in the figure corresponds to one trajectory, with $\langle a \rangle$ being the average over all He^{2+}-electron systems of the trajectory. $\langle a \rangle$ is plotted against the laser energy W_{abs} absorbed by the nanoplasma. Given are the results for three MD trajectory sets for different NIR pulse peak intensities and numbers of He*s, each set consisting of about 10 trajectories, which are extended to 3 ps to allow the nanoplasma to expand: 5×10^{13} Wcm^{-2} with $n^* = 20$ (blue), 1×10^{14} Wcm^{-2} (orange) and 2×10^{14} Wcm^{-2} (green), the latter two data sets for $n^* = 14$. The data points depend nearly linearly on W_{abs}. For a given constant pulse peak intensity and a fixed number of He*'s, W_{abs} varies considerably, being determined by the random instant at which the ionization avalanche is triggered during the laser pulse, as shown by Heidenreich *et al.*[13] We note that the simulated electron orbits are quite excentric with excentricities ranging between 0.2 and 0.9 (not shown).

When the ignition instant occurs early, the nanoplasma absorbs more laser energy. The trend of increasing $\langle a \rangle$ with increasing W_{abs} is in qualitative agreement with the experimental observation that the fluorescence yield from higher-lying states of He^{+*} increases with increasing laser intensity, see figure S3. Naturally, the classical simulation does not allow for assignment of specific quantum numbers. Nevertheless, to relate to the experiment, $\langle a \rangle$ can be compared to the expectation value of the radii of He^{+*} orbitals, $\langle r \rangle = 3a_0n^2/4$ (assuming the orbital angular momentum quantum number $\ell = 0$), that is $\langle r \rangle = 1.6$ Å for $n = 2$, $\langle r \rangle = 3.6$ Å for $n = 3$, $\langle r \rangle = 6.4$ Å for $n = 4$, etc. Here, $a_0 = 0.53$ Å is the Bohr radius.

The clear correlation of $\langle a \rangle$ with W_{abs} can be rationalized as follows: When a nanoplasma ignites, nearly all He atoms are doubly ionized in the "inner-ionization" state at short times; however, when the nanoplasma expands, it rapidly cools and electrons recombine with the ions. If the plasma is highly energetic (large W_{abs} values, high electron energies, fully doubly ionized He atoms), then the nanoplasma expands fast and electrons recombining with He^{2+} dications are trapped in high Rydberg states. At lower W_{abs}, the expansion is slower, allowing for collisions between electrons and He ions in the expanding cloud. This causes electrons to be de-excited into lower-lying states, as the effective cross sections for electron-He^{+*} collisions are large for high principal quantum numbers of He^{+*} states, n.[14] In case of

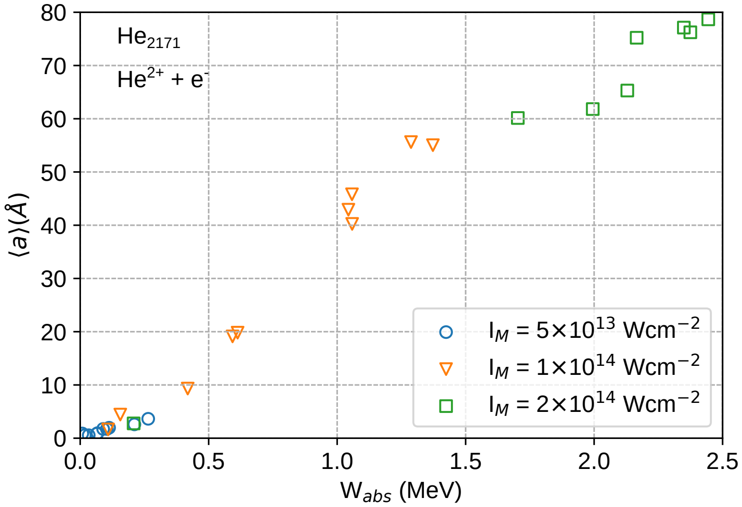

Figure S6: The average semimajor axis ($\langle a \rangle$) of the electrons in their Kepler ellipses around their central He^{2+} dications as a function of laser energy absorbed by the nanoplasma, W_{abs}. Every data point in the figure corresponds to one trajectory, with $\langle a \rangle$ being the semimajor axis of the classical electron orbits averaged over all He^{2+}-electron systems of the trajectory.

incomplete ionization of He atoms in the nanoplasma at small W_{abs} values, the average electron kinetic energy is lower. Thus, electron-He collisions by trend lead to impact excitation of lower states of He* and He^{+*}. In contrast, in energetic plasmas electron-He collisions rapidly lead to complete double ionization of He atoms, and bound excited states are populated exclusively by electron-ion recombination in the expansion phase of the nanoplasma. In our experiments, probably both mechanisms of populating excited states in He* and He^{+*} contribute.

This interpretation is supported by our experimental findings for variable droplet size and variable NIR intensity, see figures S2 and S3. Both for small droplets (*i. e.* droplet radius $R = 5$, 7 nm) and for low NIR intensity (*i. e.* at pulse energy 185 μJ), $W_{\rm abs}$ is low and, consequently, the relative fluorescence yield from the lowest excited state $n = 2$ is highest.

References

[1] A. Heidenreich, I. Infante and J. M. Ugalde, *New J. Phys.*, 2012, **14**, 075017.

[2] A. Heidenreich, B. Grüner, M. Rometsch, S. R. Krishnan, F. Stienkemeier and M. Mudrich, *New J. Phys.*, 2016, **18**, 073046.

[3] A. Heidenreich, I. Last and J. Jortner, *J. Chem. Phys.*, 2007, **127**, 074305.

[4] M. V. Ammosov, N. B. Delone and V. P. Krainov, *Sov. J. Exp. Theor. Phys.*, 1986, **64**, 1191.

[5] F. A. Ilkov, J. E. Decker and S. L. Chin, *J. Phys. B: At. Mol. Opt. Phys.*, 1992, **25**, 4005.

[6] W. Lotz, *Z. Phys.*, 1967, **206**, 205–211.

[7] T. Fennel, L. Ramunno and T. Brabec, *Phys. Rev. Lett.*, 2007, **99**, 233401.

[8] M. Mudrich, A. C. LaForge, A. Ciavardini, P. O'Keeffe, C. Callegari, M. Coreno, A. Demidovich, M. Devetta, M. D. Fraia, M. Drabbels, P. Finetti, O. Gessner, C. Grazioli, A. Hernando, D. M. Neumark, Y. Ovcharenko, P. Piseri, O. Plekan, K. C. Prince, R. Richter, M. P. Ziemkiewicz, T. Möller, J. Eloranta, M. Pi, M. Barranco and F. Stienkemeier, *Nat. Commun.*, 2020, **11**, 112.

[9] A. C. LaForge, J. D. Asmussen, B. Bastian, M. Bonanomi, C. Callegari, S. De, M. D. Fraia, L. Gorman, S. Hartweg, S. R. Krishnan, M. F. Kling, D. Mishra, S. Mandal, A. Ngai, N. Pal, O. Plekan, K. C. Prince, P. Rosenberger, E. A. Serrata, F. Stienkemeier, N. Berrah and M. Mudrich, *Phys. Chem. Chem. Phys.*, 2022, **24**, 28844–28852.

[10] D. Meschede and D. Meschede, *Optics, Light and Lasers: The Practical Approach to Modern Aspects of Photonics and Laser Physics*, Wiley-VCH, Weinheim, 2nd edn, 2007.

[11] A. Heidenreich and J. Jortner, *J. Chem. Phys.*, 2011, **134**, 074315.

[12] D. S. Peterka, J. H. Kim, C. C. Wang, L. Poisson and D. M. Neumark, *J. Phys. Chem. A*, 2007, **111**, 7449–7459.

[13] A. Heidenreich, B. Grüner, D. Schomas, F. Stienkemeier, S. R. Krishnan and M. Mudrich, *J. Mod. Opt.*, 2017, **64**, 1061–1077.

[14] Yu. Ralchenko, R. K. Janev, T. Kato, D. V. Fursa, I. Bray and F. J. de Heer, *At. Data Nucl. Data Tables*, 2008, **94**, 603–622.
